# Small Lesion Size Is Associated with Sleep-Related Epilepsy in Focal Cortical Dysplasia Type II

**DOI:** 10.3389/fneur.2018.00106

**Published:** 2018-02-28

**Authors:** Bo Jin, Wenhan Hu, Linmei Ye, Balu Krishnan, Thandar Aung, Stephen E. Jones, Imad M. Najm, Andreas V. Alexopoulos, Kai Zhang, Junming Zhu, Jianguo Zhang, Meiping Ding, Zhong Chen, Shuang Wang, Zhong Irene Wang

**Affiliations:** ^1^Department of Neurology, Epilepsy Center, Second Affiliated Hospital, School of Medicine, Zhejiang University, Hangzhou, China; ^2^Epilepsy Center, Cleveland Clinic, Cleveland, OH, United States; ^3^Department of Neurosurgery, Beijing Neurosurgical Institute, Beijing Tiantan Hospital, Capital Medical University, Beijing, China; ^4^Department of Diagnostic Radiology, Mellen Imaging Center, Cleveland Clinic, Cleveland, OH, United States; ^5^Department of Neurosurgery, Epilepsy Center, Second Affiliated Hospital, School of Medicine, Zhejiang University, Hangzhou, China; ^6^Department of Pharmacology, Key Laboratory of Medical Neurobiology of the Ministry of Health of China, Zhejiang Province Key Laboratory of Neurobiology, College of Pharmaceutical Sciences, Zhejiang University, Hangzhou, China

**Keywords:** focal cortical dysplasia type II, sleep-related epilepsy, MRI, volume, location

## Abstract

**Objective:**

To investigate the neuroimaging and clinical features associated with sleep-related epilepsy (SRE) in patients with focal cortical dysplasia (FCD) type II.

**Methods:**

Patients with histopathologically proven FCD type II were included from three epilepsy centers. SRE was defined according to the video EEG findings and seizure history. Cortical surface reconstruction and volume calculation were performed using FreeSurfer. The lesions were manually delineated on T1 volumetric MRI using the ITK-SNAP software. The lesion volumes were normalized by the intracranial volume of each patient. The lesions were classified as small or large by placing a threshold based on quantitative (whether the lesion was detected on MRI report) and qualitative (volume) criteria.

**Results:**

A total of 77 consecutive patients were included. Of them, 36 had SRE and 41 had non-SRE. An earlier age of epilepsy onset, high seizure frequency, regional interictal EEG findings, and favorable surgical outcome were characteristic in both groups. Small lesions were defined as those having a volume <3,217 mm^3^. In total, 60.9% of the patients with SRE (25/41) had small FCD lesion, which was significantly higher than the non-SRE group (9/34, 26.5%, *p* = 0.005). Small lesion size was the only predictor significantly associated with SRE in the overall type II group by multivariate analyses (*p* = 0.016). Although the proportion of patients who had frontal FCD and SRE was higher than non-frontal FCD (54.5 vs. 27.3%, *p* = 0.043), the relationship between SRE and lesion location was not confirmed by multivariate analysis. Thalamic volume and seizure semiology were not statistically different between the SRE and non-SRE group. The significant association between lesion size and SRE was reproducible in type IIb and IIa subgroups.

**Significance:**

SRE is common in patients with FCD type II. Small FCD type II lesions are significantly associated with SRE. Although our findings cannot be applied to the entire spectrum of SRE, potential existence of small FCD lesions should be considered when evaluating patients with SRE, and utilization of all other supportive electroclinical information for lesion detection is highly desirable.

## Introduction

Sleep-related epilepsy (SRE), described as most seizures occurring during sleep (1–3), represents up to 12% of patients with epilepsy. Most of the patients with SRE have focal epilepsy (4). SRE may be an independent risk factor for sudden unexpected death in epilepsy (SUDEP) (5). Sleep and epilepsy have reciprocal influences on each other: seizures occurring during sleep will significantly reduce sleep quality (6); meanwhile, sleep–wake cycle can be associated with seizures in some patients, seizure type such as tonic, tonic–clonic, automotor, and hypermotor seizures occurred more frequently in sleep than daytime (7).

Focal cortical dysplasia (FCD) type II is a major cause of pharmacoresistant extra-temporal epilepsy in patients undergoing epilepsy surgery (8, 9). One previous study showed the existence of FCD type II increased the risk of SRE with respect to other histopathological substrates, regardless of its location (10). Another study reported that SRE occurred more frequently in patients with MRI-negative FCD type II than the MRI-positive group (11). In addition to the lesion itself, there is evidence that the thalamus might play a critical role in the occurrence of SRE. The sleep–wake process modulates the electrophysiological activity generated by FCD type II, as revealed by intracranial EEG recordings, suggesting there is an interaction between seizure activity and the thalamocortical networks (4, 12).

In this study, we investigate whether the patients with SRE demonstrates specific neuroimaging or clinical features compared to the non-SRE subgroup in a cohort of patients with histopathologically proven FCD type II. We hypothesize that there are three potential factors influencing the occurrence of SRE: size of lesion, spatial/lobar distribution of lesion, and volume of thalamus. In this study, we set out to test the significance of these three factors in the overall FCD type II group and IIa/IIb subgroups.

## Materials and Methods

### Patient and SRE

We retrospectively reviewed the patients who underwent surgery and had histopathologically confirmed FCD type II from three different epilepsy centers: Second Affiliated Hospital of Zhejiang University (SAHZU), the Beijing Tiantan Hospital of Capital Medical University (BTH), and Cleveland Clinical Foundation (CCF). The enrolled patients were consecutively admitted in SAHZU from March 2013 to June 2016, BTH from November 2014 to April 2016, and CCF from August 2008 to September 2015. This study was approved by the institutional review board ethical guidelines of three hospitals (SAHZU, BTH, and CCF).

Only patients who underwent long-term video EEG (VEEG) monitoring and had been followed-up for more than 1 year after surgery were included. Patients younger than 5 years of age were excluded. All clinical, neuroimaging, EEG data, and surgical outcome were collected. Seizure frequency was categorized as high frequency (>1 seizures/day) or low frequency (≤1 seizures/day). Interictal epileptiform discharges and ictal EEG pattern were classified as normal (no EEG change), regional (appearing exclusively over a single lobe or in two contiguous regions in the same hemisphere, such as centroparietal discharges), or non-regional (e.g., multilobar, hemispheric, or generalized). Patients with multiple ictal EEG patterns (e.g., multilobar or non-lateralized patterns) were considered non-regional. All patients’ MRI were classified as “MRI-negative” or “MRI-positive” by official radiology report. The location of the lesion was classified into frontal lobe, temporal lobe, posterior quadrant, and insular/opercular region. The surgical outcome was determined according to Engel’s classification scheme (13). The patients were classified as seizure free if they maintained with an Engel score of Class I at their last follow-up. The diagnosis and classification of FCD were made by dedicated neuropathologists from each hospital, according to the ILAE guidelines (14). FCD type II was defined as an isolated lesion showing cortical dyslamination and dysmorphic neurons without (type IIa) or with balloon cells (type IIb).

Sleep-related epilepsy was defined as more than 90% of patients’ total seizures occurring during sleep (1–3). The circadian distribution of seizures was determined by description from patients and their family, and further confirmed by the long-term VEEG monitoring. All 77 patients underwent scalp VEEG recordings for at least 24 h (ranging from 1 to 7 days) using 10–20 international system. Additional scalp electrodes such as sphenoidal electrodes were used if necessary. During the long-term VEEG monitoring, patients were allowed to choose their sleep and awake times. Patients and their family were encouraged to press the seizure alarm for suspicious events. All recorded clinical seizures during monitoring were independently re-analyzed by two epileptologists, to assess the relationship of seizures to wakefulness and sleep. If their results were discordant, a final consensus was reached after discussion. Non-habitual seizures and subclinical seizures were excluded.

### MRI Acquisition

Patients from SAHZU were scanned on a 3.0-T MRI scanner (MR750, GE Healthcare, USA) including 3D T1 sagittal brain volume imaging (BRAVO) sequence (TR/TE = 8.2/3.2 ms, TI = 450 ms, flip angle = 12°, slice thickness = 1 mm, no gap, matrix = 256 × 256). Patients from BTH were scanned on a 3.0-T Siemens Verio scanner (Siemens Medical system, South Iselin, NJ, USA) including 3D T1 sagittal magnetization prepared rapid gradient echo sequence (MPRAGE) (TR/TE = 1,900/2.53 ms, TI = 900 ms, flip angle = 12°, slice thickness = 1 mm, no gap, matrix = 256 × 256). Patients from CCF were performed on a 3.0-T Siemens Trio scanner (Erlangen, Germany) including 3D T1 coronal MPRAGE (TR/TE = 1,860/3.4 ms, TI = 1,100 ms, flip angle = 10°, slice thickness = 0.94 mm, no gap, matrix = 256 × 256).

### Image Processing and Analysis

The T1-weighted 3D MRI images were processed with the FreeSurfer software (v.5.3.0^1^) (15–17). Intracranial volume (ICV) and thalamus were segmented automatically by FreeSurfer, and segmentation results for each subject were visually reviewed independently by an experienced user (Bo Jin) for segmentation errors prior to further analysis. In case of inaccuracies, manual editing was performed.

**Figure 1:**
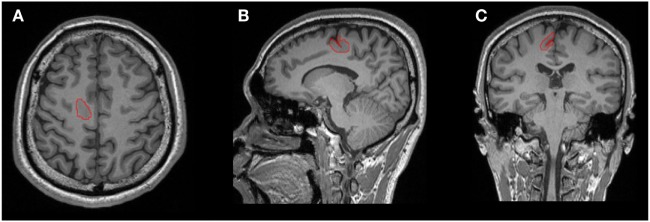
One example of manual lesion labeling using the ITK-SNAP software. Lesion volume was calculated based on the 3D T1 volumetric sequence. **(A)** Axial view; **(B)** coronal view; **(C)** sagittal view.

The lesions were manually labeled by an experienced user (Bo Jin) based on the T1-weighted 3D MR images (including axial, coronal, and sagittal) aided by FLAIR and other T2-weighted images. Lesion segmentation was performed using the ITK-SNAP software (v.3.4.0^2^; Figure 1) (18).

To classify lesion to the small and large categories, we first obtained qualitative information based on the initial MRI report (positive or negative), with the assumption that negative MRI indicated overlooked therefore small lesions. We then used a quantitative criterion based on a threshold defined by volume calculation (detailed in the next section). Lesions with a volume below this threshold were considered small lesions for further analyses.

Consistent with methods proposed by Besson et al. (19), entropy was used to calculate the threshold. This approach maximized the “information gain,” which was the gain in homogeneity after thresholding. The entropy of our dataset (*D*) was calculated with following formula:

Entropy(D)=−plog2 p−(1−p)log2(1−p)

where *p* is the number of positive MRI reports divided by total number of MRI reports.

Our dataset was divided into two sets by setting a threshold (*T*), *D*1, and *D*2. *D*1 represented the lesion volumes smaller than *T* and *D*2 represented the lesion volumes larger than *T*. The information gain was defined by the following formula:

Information Gain(D,T)=Entropy(D)−[Entropy(D1)−Entropy(D2)].

Using this threshold, the probabilities of the events “negative MRI report if smaller than *T*” and “positive MRI report if larger than *T*” were maximized simultaneously.

To correct for volume differences due to different head sizes, the lesion volumes and thalamic volumes were normalized by the total brain volume of each individual: normalized lesion or thalamic volume = lesion volume or thalamic volume × mean intracranial volume (ICV) of the overall cohort/individual ICV. For the thalamus, the side of lesion was defined as the ipsilateral or contralateral to the FCD lesion.

### Statistical Analysis

Descriptive statistics were used for each variable. The data were analyzed using the Wilcoxon rank-sum, chi-square, and Fisher’s exact tests to compare patients with SRE to those with non-SRE. Variables with a significance level of 0.1 on univariate analysis were then entered in a multivariable logistic regression model with statistical significance set at the 0.05 level.

For volumetric analysis, group differences were analyzed using analysis of covariance (ANCOVA) on age, gender, ICV, and a *post hoc* Bonferroni correction was further applied to correct for multiple comparisons. The statistical significance was set to *p* < 0.017 (0.05/3). The associations between the normalized thalamic volumes and lesion volumes were analyzed using partial correlations controlling for the effect of age and gender (*p* < 0.05). To correct for non-normal distribution, the normalized lesion volumes were log-transformed.

## Results

### Patient Characteristics

A total of 77 consecutive patients were included (17 patients from SAHZU, 17 patients from BTH, and 43 patients from CCF). Most patients had onset of epilepsy at an early age, high seizure frequency, and regional EEG findings (Table 1). Among the 77 patients, 35 patients underwent intracranial EEG evaluation (stereo-EEG or subdural electrodes). The mean age at the time of MRI scan, the mean age at seizure onset, and the mean epilepsy duration was 20.1 ± 12.9 (range 5–58) years, 6.9 ± 6.7 (0.2–35) years, and 157.5 ± 135.9 (range 4–540) months, respectively. Of them, 36 (46.7%) were SRE and 41 (53.3%) were non-SRE. The two groups showed similar clinical profiles (Table 1). Only two patients presented with epileptic spasms, which later changed into focal seizures. The percentage of SRE was similar in patients with secondary generalized tonic–clonic seizures (SGTCS, 16/32 = 50%) and patients with only focal seizures (20/45 = 44.4%).

**Table 1:** Comparison of clinical data in sleep-related FCD type II and non-sleep-related FCD type II.

Clinical characteristics	Patients with SRE (*N* = 36)	Patients with non-SRE (*N* = 41)	*p*-Value
Male, *N* (%)	20 (55.6)	23 (56.1)	0.962
Age at onset, years	7.8 (0.2–35)	6.1 (0.2–17)	0.693
Age at the MRI, years	21.8 (5–58)	18.5 (5–57)	0.257
Duration of epilepsy, months	169.0 (4–540)	147.4 (12–480)	0.489
Febrile seizures	2 (5.6%)	2 (4.9%)	1.000
Family history	2 (5.6%)	2 (4.9%)	1.000
Head trauma	0	0	–
CNS infection	0	3 (7.3%)	0.243
Perinatal adverse events	1 (2.8%)	2 (4.9%)	1.000
Status epilepticus	1 (2.8%)	1 (2.4%)	1.000
Auras	17 (47.2%)	24 (58.5%)	0.321
SGTCS	16 (44.4%)	16 (39.0%)	0.630
Mean number of seizure type	1.4 (1–2)	1.5 (1–3)	0.242
Low seizure frequency	11 (30.6%)	17 (41.5%)	0.321
MRI findings (positive)	23 (63.9%)	32(78.0%)	0.170
Left side of FCD	13 (36.1%)	22 (53.7%)	0.123
BOSD	15 (41.7%)	14 (34.1%)	0.638
FCD type IIb	26 (72.2%)	35 (85.4%)	0.173
IEDs (normal/regional/non-regional)	6/18/10	6/25/9	0.701
Ictal EEG (normal/regional/non-regional)	4/18/12	1/27/12	0.209
Invasive EEG	22 (61.1%)	19 (46.3%)	0.254
Seizure-free (Engel I)	29 (80.6%)	29 (70.7%)	0.428

*CNS, central nervous system; SGTCS, secondary generalized tonic–clonic seizures; FCD, focal cortical dysplasia; BOSD, bottom-of-sulcus cortical dysplasia; IEDs, interictal epileptiform discharges; SRE, sleep-related epilepsy*.

The initial MRI reports were positive in 55 patients (71.4%). After re-reviewing the MRI with integrated information of functional imaging and electroclinical data, MRI was considered as positive in 75 (97.4%). The remaining two patients whose MRI lesion was strictly non-visible (one lesion was located in the frontal lobe, the other in the posterior quadrant) were not included in the volumetric analyses (as no volume can be manually delineated on the MRI). Interestingly, both patients had FCD type IIa and SRE.

**Figure 2:**
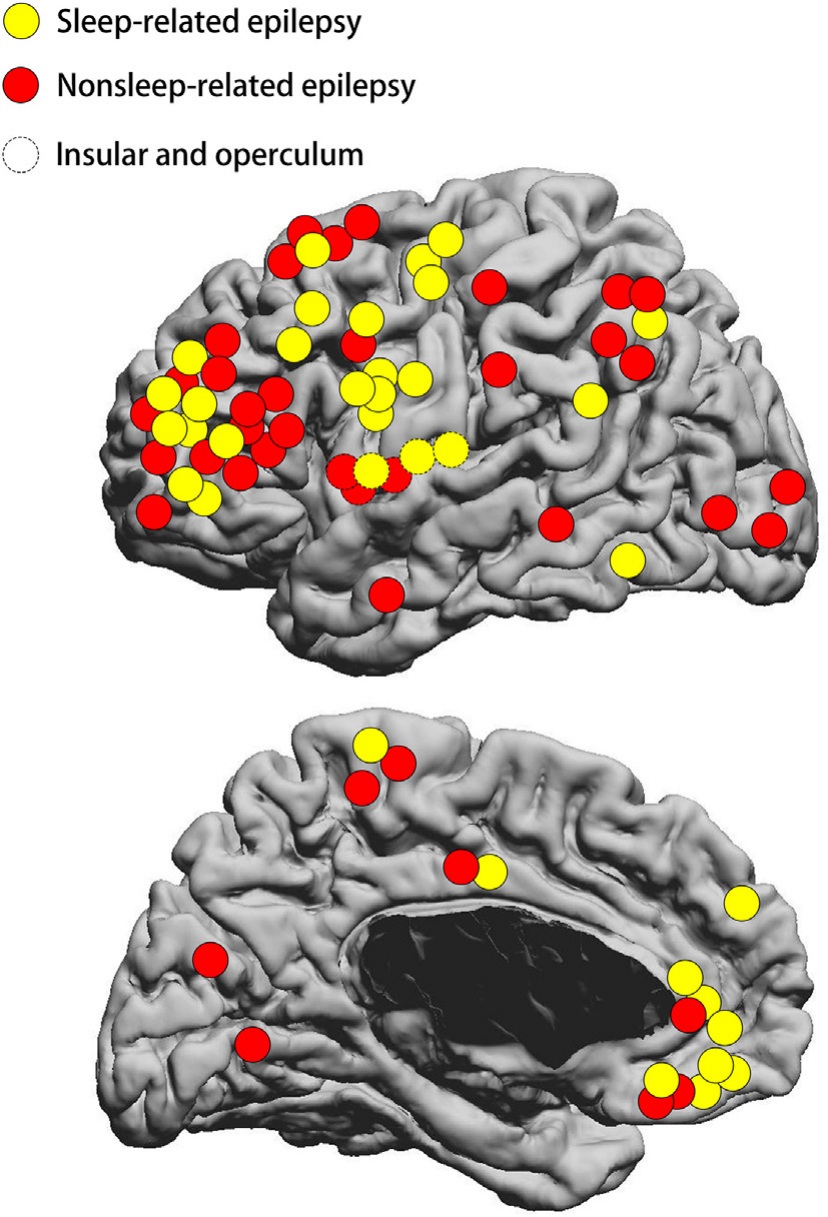
Schematic diagram showing the location distribution of the lesions included in our study. Yellow circles represent SRE; red circles represent non-SRE. Dashed circles represent focal cortical dysplasia (FCD) type II on the insular and operculum. SRE, sleep-related epilepsy. The location of FCD lesion was not statistically different between the SRE and non-SRE group.

The FCD type II lesions were located in the frontal lobe in 55 patients, temporal lobe in 2 patients, posterior quadrant in 14 patients, and insular/opercular region in 6 patients (Figure 2). The frontal FCD type II group (30/55 = 54.5%) had a higher rate of SRE compared to non-frontal FCD type II (6/22 = 27.3%, *p* = 0.043). Meanwhile, the percentage of small FCD lesion was higher in the frontal lobe (35/54 = 64.8%) than the other regions (6/21 = 28.6%, *p* = 0.009). In addition, most patients with FCD located in the posterior quadrant had non-SRE (11/14 = 78.6%), while a smaller percentage of non-SRE patients (30/63 = 47.6%, *p* = 0.043) had FCD outside the posterior quadrant. At the same time, the percentage of large FCD lesion was higher in the posterior quadrant (10/13 = 76.9%) than the other regions (31/62 = 50%). Surgical outcome was favorable in both SRE and non-SRE groups. Engel class I was obtained in 29 patients with SRE (80.6%) and 29 patients with non-SRE (70.7%) which was not different. Sixteen patients had FCD type IIa and 61 patients had FCD type IIb.

### Lesion and Thalamus Volumes

**Figure 3:**
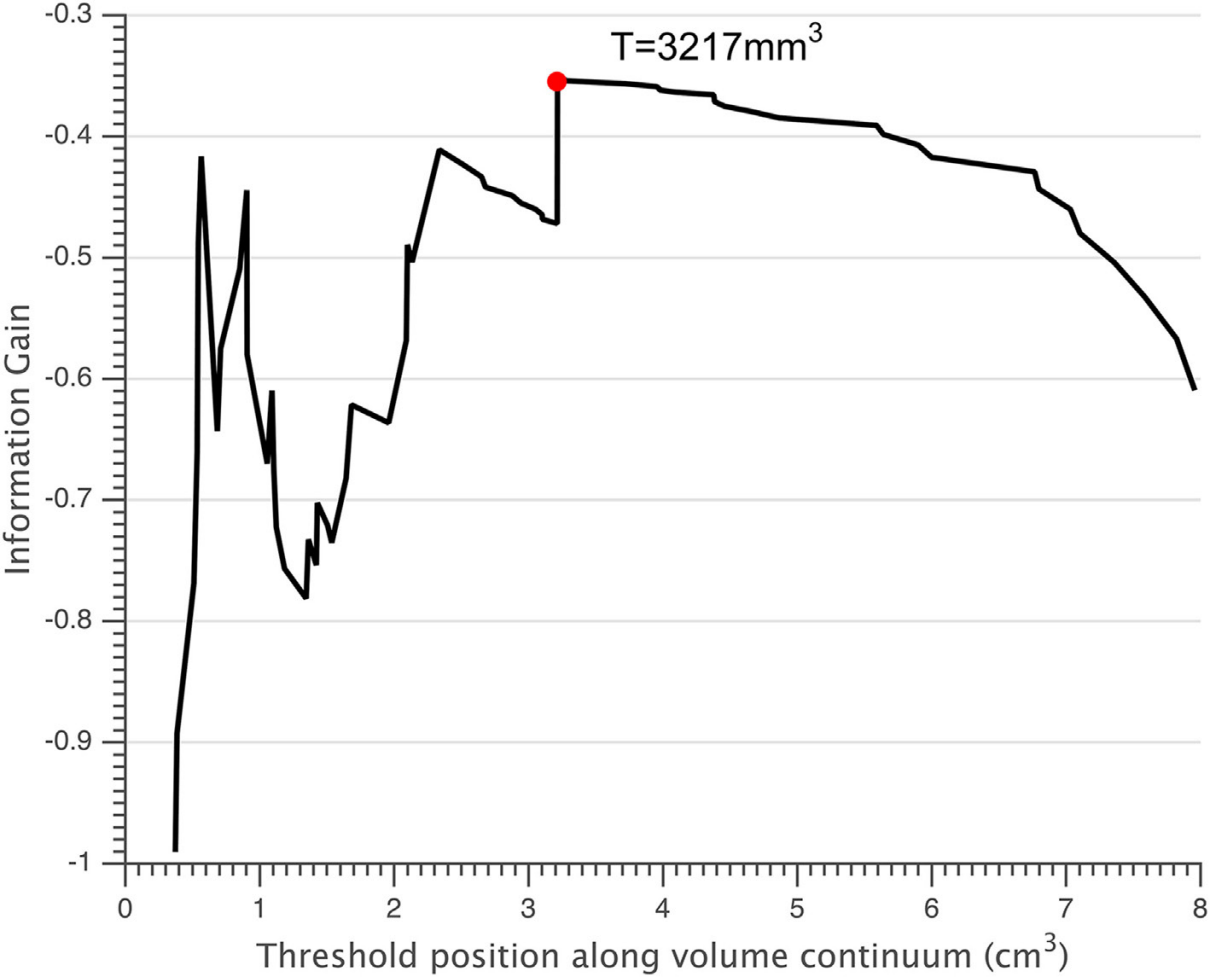
Small lesions were defined as those having a volume below the threshold *T* = 3,217 mm^3^ (red circle).

Normalized volumes of the lesions and thalamus were displayed in Table 2. Small lesions were defined as those with a volume smaller than the threshold of 3,217 mm^3^ (Figure 3). According to this threshold, 41 of 75 FCD lesions were classified as small lesions. In the small lesion group, 21 out of 41 lesions (51.2%) were located at the bottom of sulcus (bottom of sulcus cortical dysplasia, BOSD), whereas only 8 out of 34 patients with large lesion were BOSD (23.5%, *p* = 0.018). Of 41 patents with small lesion, 25 (25/41 = 60.9%) patients had SRE which was significantly high compared to the large lesion group (9/34 = 26.5%, *p* = 0.005). Accordingly, the percentage of the patients with SRE and small lesion was higher than the non-SRE group (25/34 = 73.5% vs. 16/41 = 39.0%, *p* = 0.005).

**Table 2:** Normalized volumes of FCD type II lesion and thalamus.

	Patients with SRE (*N* = 36)	Patients with non-SRE (*N* = 41)	*p*-Value
FCD type II (mm^3^)^a^	1,536.78 (290.69–9,635.76)	3,834.23 (385.40–29,989.71)	
Log10 (FCD type II)^a^	3.28 ± 0.42	3.54 ± 0.40	0.014*

**Thalamus (mm3)**			
Ipsilateral	7,432.08 ± 1,011.40	7,506.78 ± 804.39	0.792
Contralateral	7,779.10 ± 993.35	7,530.00 ± 793.92	0.279

*FCD, focal cortical dysplasia; SRE, sleep-related epilepsy*.

*Significantly different estimates.

*^a^34 patients with SRE and 41 patients with non-SRE were included in the volume of FCD type II; a *post hoc* Bonferroni correction was further applied to correct for multiple comparisons; the statistical significance was set to *p* < 0.017 (0.05/3)*.

To correct for non-normal distribution, the normalized volumes of FCD type II were log-transformed. Similarly, ANCOVA showed that SRE was significantly associated with smaller lesion volume when controlling with age and gender (Table 2, *p* = 0.014). However, the thalamic volume was not different between the two groups. The volumes of FCD type II showed no correlation with the volume of ipsilateral and contralateral thalamus, controlling for age, gender, and ICV. Age, gender, lesion size, and lesion location were entered into logistic regression analysis, which showed small lesion was independently associated with SRE (Table 3). For patients with frontal lobe FCD type II, logistic regression analysis showed that the risk factor for SRE was small lesion size [*p* = 0.04, risk factor = 3.73, 95% confidence intervals (CIs) = 1.06–13.04].

**Table 3:** Variables correlating with SRE after applying logistic regression analysis.

Predictor	Risk ratio	95% CI	*p*-Value
Age	0.97	0.94–1.02	0.21
Gender	0.50	0.16–1.54	0.23
Small lesion	3.67	1.28–10.57	0.016*
Frontal lobe^a^	1	–	–
Temporal lobe	1.74E+9	0	0.99
Insular/opercular region	0.76	0.13–4.63	0.77
Posterior quadrant	4.14	0.78–21.99	0.1

*SRE, sleep-related epilepsy; CI, confidence interval*.

*Significantly different estimates.

*^a^Frontal lobe served as control*.

### Subgroup Analysis

The percentage of SRE was similar in patients with FCD type IIb (26/61 = 42.6%) and patients with FCD type IIa (10/16 = 62.5%).

In addition, the distribution of lesion was not different between FCD type IIb and FCD type IIa (frontal lobe, 42 IIb and 13 IIa; temporal lobe, 2 IIb and 0 IIa; insular/opercular region, 6 IIb and 0 IIa; posterior quadrant, 11 IIb and 3 IIa; *p* = 0.498), although FCD type IIa subgroup had fewer patients.

According to the volumetric threshold, 33 of 61 patients (54.1%) with FCD type IIb and 8 of 14 patients (57.1%) with FCD type IIa were classified as having small lesion. For patients with FCD type IIb, although the percentage of the patients with SRE and small lesion was higher than the non-SRE group (18/33 = 54.5% vs. 8/28 = 28.6%), it did not reach statistical significance. Similar to the entire FCD type II group, logistic regression analysis showed that small type IIb lesion was independently associated with SRE (*p* = 0.044, risk factor = 3.00, 95% CIs = 1.03–8.73). In the patients with FCD type IIa, seven out of eight patients with small lesion (87.5%) had SRE, whereas only one out of six patients (16.7%) with large lesion had SRE (Table 4, *p* = 0.026). Logistic regression analysis cannot be applied to this group due to the small number of patients. Detailed information on this subgroup analysis can be found in Table 4.

**Table 4:** Lesion volumes, subtypes, and bottom sulcal localization in patients with SRE and non-SRE.

	FCD type IIa (*N* = 16)			FCD type IIb (*N* = 61)		
	Patients with SRE (*N* = 10)	Patients with non-SRE (*N* = 6)	*p*-Value	Patients with SRE (*N* = 26)	Patients with non-SRE (*N* = 35)	*p*-Value
Lesion volume^a^ (mm^3^)	2,409.89 (535.53–7,958.23)	4,468.72 (3,216.62–6,541.85)		3,161 (290.69–9,635.76)	5,327.88 (385.40–29,989.71)	
Log10 (lesion volume)^a^	3.21 (2.73–3.90)	3.63 (3.51–3.28)	0.018*	3.31 (2.46–3.98)	3.52 (2.59–4.48)	0.039*
Small lesion^a^	7 (87.5%)	1(16.7%)	0.026*	18 (69.2%)	15 (42.9%)	0.068
BOSD^a^	4 (50%)	2 (33.3%)	0.533	11(42.3%)	12 (34.3%)	0.598

*^a^Two FCD type IIa patients whose MRI lesion was strictly non-visible were not included in this analysis*.

*Significantly different estimates.

*BOSD, bottom-of-sulcus cortical dysplasia; FCD, focal cortical dysplasia; SRE, sleep-related epilepsy*.

## Discussion

Sleep-related epilepsy is common in patients with FCD type II and occurred in 46.7% of the patients included in our study. This percentage was similar to the previous findings that about 50% patients with FCD type II had SRE (10, 11, 20). Our volumetric analysis showed a correlation between SRE and smaller lesion size which was independent of lesion location and thalamic volume. Our findings are consistent with the previous study by Chassoux et al. which found there was no correlation between SRE and the location of lesion in patients with histologically confirmed FCD type II (11). Nobili et al. studied 39 drug-resistant epilepsy patients who were seizure-free after resective surgery, and reported Taylor’s FCD (which is equivalent to FCD type II by the 2011 ILAE FCD classification system) was associated with SRE irrespective of the location of lesion (10). Our study added to the literature in the following aspects: (1) we showed that small lesion size was significant associated with SRE in both the IIa and the IIb subgroups. (2) We used a quantitative threshold to define small and large lesions, and showed that patients with small lesions were about four times more likely to have SRE compared to those with large lesions. Note that both SRE group and non-SRE group similarly presented with an earlier age of epilepsy onset, high seizure frequency, regional interictal EEG findings, and favorable surgical outcome, which is in accordance with previous studies focusing on FCD type II (2, 8, 9, 21). These results suggest that patients with SRE and electroclinical data suggestive of FCD may have small lesions, and they require a more thorough imaging investigation for lesion detection. Once the small lesions are found, these patients can become excellent surgical candidates as revealed by the high seizure-free rates in our cohort (80.6%).

One might raise a reasonable question whether the higher risk of SRE in patients with FCD type II is related to a frontal predominance of FCD distribution. To address this question, we examined the subgroup of patients with frontal FCD, and multivariate analysis showed that small lesion was an independent risk factor for SRE in this subgroup.

In accordance with a previous study by Besson et al. (19), our current study showed that the majority of small FCD lesions were located at the bottom of sulcus. Recently, Harvey et al. reported that 13 out 32 (40.6%) patients with BOSD had seizures occurring exclusively from sleep (22). Similarly, in our present study, 51.7% of 29 patients with BOSD were SRE. However, we did not find any statistically significant association between the BOSD and SRE in our cohort. Moreover, neither the FCD type IIa nor FCD type IIb subgroup showed a significant association between BOSD and SRE.

Chassoux reported that SRE occurred more frequently in patients with MRI-negative FCD type II than the MRI-positive group (11). In their cohort of FCD type II, 40% of the patients were defined as MRI-negative epilepsy. However, in our study, only 2 out of the 77 patients with FCD type II was MRI negative. The discrepancy of the ratio of MRI-negative cases in the two study is likely due to the advance in neuroimaging techniques in recent years. First, the three epilepsy centers in our study routinely performed brain MRI using epilepsy protocol based on 3-T scanner, which is more superior in detecting subtle lesions (23); second, the MRI-PET co-registration was routinely done for evaluation of these patients; third, other functional neuroimaging techniques such as ictal SPECT or magnetoencephalography are performed if necessary. Furthermore, evaluating the MRI by a multi-disciplinary team further increases the sensitivity to identify epileptogenic lesions.

During wakefulness, the most common intracranial interictal activity (subcontinuous spikes and polyspikes) produced by intralesional FCD type II might indicate a state of reduced possibility for seizure generation (4, 12, 24–26). These patterns would be replaced by frequent short bursts of low voltage fast discharges during NREM sleep, which might relate to a reduced modulation of the FCD type II by the surrounding cortex and therefore might play a pivotal role in seizure initiation (4, 27–29). We speculate that perhaps small FCD type II lesion, which contained smaller mass of dysplastic neurons, might not be able to generate seizure by itself during wakefulness, had to recruit perilesional cortex and utilize the sleep rhythm to generate seizure ep rhythm; therefore, seizures could occur equally easily during wakefulness and sleep (4, 11). Large lesion, on the other hand, due to its lager mass of dysplastic neurons might not need the help of perilesional and sleep rhythm; therefore, seizures could occur equally easily during wakefulness and sleep (4, 11).

Several studies using intracranial EEG suggested that the dysplastic tissues were influenced by the thalamocortical circuit (12, 25, 26). Despite the suspicion that thalamus plays an important role in the generation of SRE, no study has investigated the thalamic volume changes in SRE. Our study is the first study investigating the thalamic volume differences in patients with FCD type II and SRE, and showed there was no difference between SRE and non-SRE in thalamic volumes. One should note that subtle subcortical structural change such as regional atrophy may not by detected by the volumetric analysis method adopted in this study. Shape analysis which can reflect subtle variations in subcortical structures can be applied as a future step (30). In our study, no correlation was found between the lesion volume and the thalamic volume either. Future study using functional imaging such as resting-state functional MRI may give us more insight regarding the functional interaction between the lesional/perilesional cortex and the thalamus in SRE.

Our study has several limitations. The number of patients with IIa and IIb FCD subtypes was unbalanced. We had 16 patients with FCD type IIa, while the majority (61 patients) had FCD type IIb. Subgroup analysis results could be influenced by this selection bias. The volume of ICV and thalamus may be influenced by the different MRI scanners with different acquisition sequences. Future study based on a large cohort of patients with FCD type II from one single epilepsy center is required to confirm our finding. Furthermore, the volume of lesion was evaluated based on the visually visible lesion on MRI, which may not include the entire extent of the microscopic lesion (31). Finally, we only included patients above 5 years of age, further studies are needed to include patients under 5 years of age to see if our findings are consistent.

## Conclusion

Our study reveals that for patients with FCD type II, there is a strong correlation between SRE and small lesion size which was independent of lesion location and thalamic volume. When evaluating patients with SRE, potential existence of small FCD type II lesions should be considered. After re-reviewing the MRI with integrated information of functional imaging and electroclinical data, the percentage of positive MRI increased by 26% in our cohort. Therefore, a thorough imaging re-review, accompanied by detailed history and other clinical data, is especially important and potentially high yield for SRE patients with an initial negative MRI.

## Ethics Statement

This study was approved by the institutional review board ethical guidelines of three hospitals (Second Affiliated Hospital of Zhejiang University; Beijing Tiantan Hospital of Capital Medical University; Cleveland Clinical Foundation), and written informed consent were obtained from all participants or their guardians after a complete description of the required measurements.

## Author Contributions

BJ: contributed to the conception, design the study, analysis of the data, interpretation of the results, and drafting the manuscript. WH: design the study, analysis of the data, and interpretation of the results. LY: revising the manuscript. BK: analysis of the data. TA: revising the manuscript. SJ: revising the manuscript. IN and AA: interpretation of the results. KZ: interpretation of the results. JMZ: interpretation of the results. JGZ: interpretation of the results. ZC: interpretation of the results and final approval of the version to be published. SW, MD, and ZW: interpretation of the results, drafting the manuscript, and final approval of the version to be published.

## Conflict of Interest Statement

The authors declare that the research was conducted in the absence of any commercial or financial relationships that could be construed as a potential conflict of interest.

## Footnotes

^1^http://surfer.nmr.mgh.harvard.edu/.

^2^www.itksnap.org/pmwiki/pmwiki.php.

## References

[1] Iber C Ancoli-lsrael S Chesson A Quan SF The AASM Manual for Scoring of Sleep and Associated Events: Rules, Terminology and Technical Specifications. 1st ed Westchester, IL: American Academy of Sleep Medicine (2007).

[2] Losurdo A Proserpio P Cardinale F Gozzo F Tassi L Mai R Drug-resistant focal sleep related epilepsy: results and predictors of surgical outcome. Epilepsy Res (2014) 108(5):953–62. 10.1016/j.eplepsyres.2014.02.016 24679947

[3] Proserpio P Cossu M Francione S Gozzo F Lo Russo G Mai R Epileptic motor behaviors during sleep: anatomo-electro-clinical features. Sleep Med (2011) 12(Suppl 2):S33–8. 10.1016/j.sleep.2011.10.018 22136897

[4] Gibbs SA Proserpio P Terzaghi M Pigorini A Sarasso S Lo Russo G Sleep-related epileptic behaviors and non-REM-related parasomnias: insights from stereo-EEG. Sleep Med Rev (2016) 25:4–20. 10.1016/j.smrv.2015.05.002 26164370

[5] Lamberts RJ Thijs RD Laffan A Langan Y Sander JW. Sudden unexpected death in epilepsy: people with nocturnal seizures may be at highest risk. Epilepsia (2012) 53(2):253–7. 10.1111/j.1528-1167.2011.03360.x 22192074

[6] Jain SV Kothare SV Sleep and epilepsy. Semin Pediatr Neurol (2015) 22(2):86–92. 10.1016/j.spen.2015.03.005 26072338

[7] Loddenkemper T Vendrame M Zarowski M Gregas M Alexopoulos AV Wyllie E Circadian patterns of pediatric seizures. Neurology (2011) 76(2):145–53. 10.1212/WNL.0b013e318206ca46 21220719

[8] Sisodiya SM Fauser S Cross JH Thom M. Focal cortical dysplasia type II: biological features and clinical perspectives. Lancet Neurol (2009) 8(9):830–43. 10.1016/S1474-4422(09)70201-7 19679275

[9] Lerner JT Salamon N Hauptman JS Velasco TR Hemb M Wu JY Assessment and surgical outcomes for mild type I and severe type II cortical dysplasia: a critical review and the UCLA experience. Epilepsia (2009) 50(6):1310–35. 10.1111/j.1528-1167.2008.01998.x 19175385

[10] Nobili L Cardinale F Magliola U Cicolin A Didato G Bramerio M Taylor’s focal cortical dysplasia increases the risk of sleep-related epilepsy. Epilepsia (2009) 50(12):2599–604. 10.1111/j.1528-1167.2009.02169.x 19519797

[11] Chassoux F Landre E Mellerio C Turak B Mann MW Daumas-Duport C Type II focal cortical dysplasia: electroclinical phenotype and surgical outcome related to imaging. Epilepsia (2012) 53(2):349–58. 10.1111/j.1528-1167.2011.03363.x 22221288

[12] Menezes Cordeiro I von Ellenrieder N Zazubovits N Dubeau F Gotman J Frauscher B. Sleep influences the intracerebral EEG pattern of focal cortical dysplasia. Epilepsy Res (2015) 113:132–9. 10.1016/j.eplepsyres.2015.03.014 25986200 PMC4451468

[13] Engel JJ Van Ness P Rasmussen T Ojrmann L Outcome with respect to epileptic seizures. In: Engel J Jr, editor. Surgical Treatment of the Epilepsies. New York: Raven Press (1993). p. 609–23.

[14] Blümcke I Thom M Aronica E Armstrong DD Vinters HV Palmini A The clinicopathological spectrum of focal cortical dysplasias: a consensus classification proposed by an ad hoc Task Force of the ILAE Diagnostic Methods Commission. Epilepsia (2011) 52(1):158–74. 10.1111/j.1528-1167.2010.02777x 21219302 PMC3058866

[15] Destrieux C Fischl B Dale A Halgren E. Automatic parcellation of human cortical gyri and sulci using standard anatomical nomenclature. Neuroimage (2010) 53(1):1–15. 10.1016/j.neuroimage.2010.06.010 20547229 PMC2937159

[16] Fischl B Salat DH Busa E Albert M Dieterich M Haselgrove C Whole brain segmentation: automated labeling of neuroanatomical structures in the human brain. Neuron (2002) 33(3):341–55. 10.1016/S0896-6273(02)00569-X 11832223

[17] Fischl B van der Kouwe A Destrieux C Halgren E Segonne F Salat DH Automatically parcellating the human cerebral cortex. Cereb Cortex (2004) 14(1):11–22. 10.1093/cercor/bhg087 14654453

[18] Yushkevich PA Piven J Hazlett HC Smith RG Ho S Gee JC User-guided 3D active contour segmentation of anatomical structures: significantly improved efficiency and reliability. Neuroimage (2006) 31(3):1116–28. 10.1016/j.neuroimage.2006.01.015 16545965

[19] Besson P Andermann F Dubeau F Bernasconi A. Small focal cortical dysplasia lesions are located at the bottom of a deep sulcus. Brain (2008) 131(Pt 12):3246–55. 10.1093/brain/awn224 18812443

[20] Noli D Bartuluchi M Gonzalez FS Kaltenmeier MC Cersosimo R Rugilo C Type II focal cortical dysplasia: electroclinical study and surgical outcome in 31 pediatric patients. Childs Nerv Syst (2013) 29(11):2079–87. 10.1007/s00381-013-2165-x 23832072

[21] Jin B Wang J Zhou J Wang S Guan Y Chen S A longitudinal study of surgical outcome of pharmacoresistant epilepsy caused by focal cortical dysplasia. J Neurol (2016) 263(12):2403–10. 10.1007/s00415-016-8274-1 27632178

[22] Harvey AS Mandeelstam SA Maixner WJ Leventer RJ Semmelroch M MacGregor D The surgically remediable syndrome of epilepsy associated with bottom-of-sulcus dysplasia. Neurology (2015) 84(20):2021–8. 10.1212/WNL.0000000000001591 25888556 PMC4442099

[23] Mellerio C Labeyrie MA Chassoux F Roca P Alami O Plat M 3T MRI improves the detection of transmantle sign in type 2 focal cortical dysplasia. Epilepsia (2014) 55(1):117–22. 10.1111/epi.12464 24237393

[24] Chassoux F Devaux B Landre E Turak B Nataf F Varlet P Stereoelectroencephalography in focal cortical dysplasia: a 3D approach to delineating the dysplastic cortex. Brain (2000) 123(Pt 8):1733–51. 10.1093/brain/123.8.1733 10908202

[25] Francione S Nobili L Cardinale F Citterio A Galli C Tassi L. Intra-lesional stereo-EEG activity in Taylor’s focal cortical dysplasia. Epileptic Disord (2003) 5(Suppl 2):S105–14. 14617429

[26] Tassi L Garbelli R Colombo N Bramerio M Russo GL Mai R Electroclinical, MRI and surgical outcomes in 100 epileptic patients with type II FCD. Epileptic Disord (2012) 14(3):257–66. 10.1684/epd.2012.0525 22963868

[27] Garbelli R Frassoni C Condorelli DF Trovato Salinaro A Musso N Medici V Expression of connexin 43 in the human epileptic and drug-resistant cerebral cortex. Neurology (2011) 76(10):895–902. 10.1212/WNL.0b013e31820f2da6 21383325

[28] Massimini M Amzica F. Extracellular calcium fluctuations and intracellular potentials in the cortex during the slow sleep oscillation. J Neurophysiol (2001) 85(3):1346–50. 10.1152/jn.2001.85.3.1346 11248006

[29] Steriade M Timofeev I Grenier F. Natural waking and sleep states: a view from inside neocortical neurons. J Neurophysiol (2001) 85(5):1969–85. 10.1152/jn.2001.85.5.1969 11353014

[30] Chung SJ Shin JH Cho KH Lee Y Sohn YH Seong JK Subcortical shape analysis of progressive mild cognitive impairment in Parkinson’s disease. Mov Disord (2017) 30(10):1447–56. 10.1002/mds.27106 28737237

[31] Bouet R Mauguiere F Daligault S Isnard J Guenot M Bertrand O The relationship between morphological lesion, magnetic source imaging, and intracranial stereo-electroencephalography in focal cortical dysplasia. Neuroimage Clin (2017) 15:71–9. 10.1016/j.nicl.2017.04.018 28491494 PMC5412109

